# IqYmune® is an effective maintenance treatment for multifocal motor neuropathy: A randomised, double‐blind, multi‐center cross‐over non‐inferiority study vs Kiovig®—The LIME Study

**DOI:** 10.1111/jns.12291

**Published:** 2018-12-11

**Authors:** Jean‐Marc Léger, Ousmane Alfa Cissé, Dario Cocito, Jean‐Marie Grouin, Haider Katifi, Eduardo Nobile‐Orazio, Rabye Ouaja, Jean Pouget, Yusuf A. Rajabally, Teresa Sevilla, Ingemar S. J. Merkies

**Affiliations:** ^1^ National Referral Center for Neuromuscular Diseases University Hospital Pitié‐Salpétrière Paris France; ^2^ Global Medical Affairs, LFB Les Ulis France; ^3^ Department of Neurosciences, Molinette Hospital Università degli Studi di Torino Torino Italy; ^4^ Department of Statistics Rouen University Rouen France; ^5^ Wessex Neurological Centre Southampton General Hospital Southampton UK; ^6^ Neuromuscular and Neuroimmunology Service, Humanitas Clinical and Research Center Milan University Milan Italy; ^7^ National Referral Center for Neuromuscular Diseases University Hospital La Timone Marseille France; ^8^ School of Life and Health Sciences, Aston Brain Centre Aston University Birmingham UK; ^9^ Neurology Department, La Fe University Hospital Centro de investigación Biomédica en red de enfermedades raras (CIBERER), University of Valencia Valencia Spain; ^10^ Maastricht University Medical Center Maastricht The Netherlands; ^11^ St. Elisabeth Hospital Willemstad Curacao

**Keywords:** clinical trial, immunoglobulin, IVIg, multifocal motor neuropathy

## Abstract

Intravenous immunoglobulin (IVIg) is the gold‐standard for maintenance treatment of multifocal motor neuropathy (MMN). This phase III, randomised, double‐blind, multi‐centre, active‐control, crossover study, aimed to evaluate the non‐inferiority of IqYmune® relative to Kiovig®, primarily based on efficacy criteria. Twenty‐two adult MMN patients, treated with any brand of IVIg (except Kiovig® or IqYmune®) at a stable maintenance dose within the range of 1 to 2 g/kg every 4 to 8 weeks, were randomised to receive either Kiovig® followed by IqYmune®, or IqYmune® followed by Kiovig®. Each product was administered for 24 weeks. The primary endpoint was the difference between IqYmune® and Kiovig® in mean assessments of modified Medical Research Council (MMRC) 10 sum score (strength of 5 upper‐limb and 5 lower‐limb muscle groups, on both sides, giving a score from 0 to 100) during the evaluation period (non‐inferiority margin of Δ = 2). A linear mixed model analysis demonstrated the non‐inferiority of IqYmune® relative to Kiovig®, independently of the covariates (value at baseline, treatment period, and treatment sequence). The estimated “IqYmune® − Kiovig®” difference was −0.01, with a 95% confidence interval (CI) −0.51 to 0.48. The number of adverse reactions (ARs) and the percentage of patients affected were similar for the two products: 39 ARs in 10 patients with IqYmune® vs 32 ARs in 11 patients with Kiovig®. No thromboembolic events nor haemolysis nor renal impairment were observed. In this first clinical trial comparing two IVIg brands for maintenance treatment of MMN, efficacy and tolerability of both brands were similar.

## 1 INTRODUCTION

Intravenous immunoglobulin (IVIg) is the gold‐standard first‐line treatment for multifocal motor neuropathy (MMN), recommended by both European Federation of Neurological Societies/Peripheral Nerve Society (EFNS/PNS) for MMN management,^1^ and European Federation of Neurological Societies (EFNS) Guidelines for the use of IVIg to treat neurological diseases.^2^ Randomised controlled trials have shown that IVIg improves muscle strength and reduces disability in MMN.^3^ Prolonged remission has been observed after IVIg therapy,^4^ but only in rare cases. In most cases, repeated courses of IVIg treatment are required to maintain the beneficial effects.

Several brands of IVIg have received approval for use for MMN in individual European countries but only one brand (Kiovig® Baxter) has received approval by the European Medicines Agency (in 2011) and the US Food and Drug Administration (in 2012).

IqYmune® is a highly purified 10% liquid preparation of normal human immunoglobulin for intravenous administration that has been shown to be effective and well‐tolerated in patients with primary immunodeficiency.^5^ It has been approved since 2015 in Europe, as a replacement therapy for primary immunodeficiency syndrome and for various types of hypogammaglobulinemia, and for immunomodulation in primary immune thrombocytopenia, Guillain‐Barré syndrome, and Kawasaki disease.

The immunomodulatory properties of IqYmune® made it worth investigating its value as a maintenance treatment for MMN. This study comparing IqYmune® with an active control was approved at a scientific advice meeting with the European Medicines Agency in January 2012.

This study aimed to determine whether IqYmune® is non‐inferior to Kiovig®, primarily based on efficacy criteria. Investigation of the safety of IqYmune® was a secondary objective.

## 2 PATIENTS AND METHODS

### 2.1 Study design and treatment

This phase III randomised, double‐blind, active‐control, crossover non‐inferiority trial was conducted at 14 sites in four countries (France, United Kingdom, Italy, and Spain). The study protocol and its amendments were approved by the Ethics Committees of all centres and were authorised by the European Medicines Agency. The study was conducted in accordance with the Declaration of Helsinki as amended in 2013 and good clinical practice. All participants provided written informed consent. The study is registered at ClinicalTrials.gov (NCT01951924) and EudraCT (2012‐001995‐12).

Participants were randomised 1:1 to two sequence groups, via a centralised interactive web response system. There was no predefined randomisation list. Instead, assignment was done dynamically using the minimisation method of Pocock and Simon^6^ to reduce the risk of imbalanced treatment sequence assignment in sites and study. In case of a treatment sequence imbalance within the site (or globally if balance was achieved within the site) a new subject was randomised with a probability of 85% to the underrepresented sequence. If sequences were balanced both within the site and globally, a new subject was randomised between sequences using a probability of 50%.

The participants allocated to sequence A initially received Kiovig® for 21 to 25 weeks (period 1) and were then treated with IqYmune® for 21 to 25 weeks (period 2). The participants in sequence B received IqYmune® during period 1, followed by Kiovig® in period 2. The evaluation period ran from 13 weeks after the initiation of either IqYmune® or Kiovig® until the end of the corresponding period.

Before shipment to investigational sites, each vial of product was covered by a masking system and was packaged in an individual box. Each vial and box was labelled with a unique identification number and all other useful information for the study except information enabling the identification of the product. Before each course, the hospital pharmacist logged in the interactive web response system and obtained the vial identification numbers for vials to be administered to the subject. The hospital pharmacist finalised the blind aspect of the vial by masking the vials' caps. The products were provided to the investigator with a masking system and infusion lines, ready for intravenous administration with a B‐Braun infusion pump (Infusomat Space). The dose and frequency of treatment were maintained at pre‐randomisation levels. The allowed range was between 1 g/kg over 1 to 3 days and 2 g/kg over 2 to 5 days every 4 to 8 weeks (±7 days). The maximum dose and frequency was 2 g/kg every 4 weeks.

After the initiation of the trial, two major modifications were made to the exclusion criteria. Participants who had previously been treated with Kiovig® could be enrolled, if they had not received Kiovig® during the last 6 months. Given the half‐life of Kiovig® in adults, this period was considered sufficiently long to limit any potential carryover effect. The potential risk of acute renal failure and renal monitoring in subjects at risk were addressed by adding a urine protein reagent strip test before the initiation of product administration and at all follow‐up visits for subjects with abnormal results for this test at screening or with a glomerular filtration rate (GFR) in the 60 to 80 mL/min/1.73 m^2^ range. The exclusion limit for GFR was decreased from 80 to 60 mL/min/1.73m^2^. An albumin‐to‐creatinine ratio >30 mg/mmol and a protein‐to‐creatinine ratio >50 mg/mmol were added as exclusion criteria.

### 2.2 Participant selection

Adult men and women (≥18 years old) were eligible for inclusion if they had been diagnosed with probable or definite MMN according to the European Federation of Neurological Societies/Peripheral Nerve Society (EFNS/PNS) 2010 guidelines,^1^ and were being treated with a stable maintenance dose, within a 15% range, of any brand of IVIg (Kiovig® excluded during the last 6 months before enrolment) at a dose between 1 g/kg over 1 to 3 days and 2 g/kg over 2 to 5 days every 4 to 8 weeks (±7 days) for at least 3 months before enrolment. The main exclusion criteria were known hypersensitivity to Ig therapy, anti‐IgA antibodies, GFR <60 mL/min/1.73 m^2^ estimated by the Modification of Diet in Renal Disease equation in adults, serum levels of alanine aminotransferase or aspartate aminotransferase >2 times the upper limit of the normal range, protein‐losing enteropathy or nephrotic syndrome, pregnancy, breastfeeding, or a history of thrombosis.

### 2.3 Outcome measures

The primary efficacy outcome was the Modified Medical Research Council (MMRC) sum score of 10 predetermined muscle groups, as described by Cats et al,^7^ during the evaluation period. Five muscle groups from the arm and five from the leg were tested (Table 1). Each muscle group was scored from 0 (paralysis) to 5 (normal strength) and 10 movements were assessed on both sides, resulting in a total score between 0 (complete paralysis) and 100 (full strength).

**Table 1:** Muscle groups tested for each MMRC sum score

Muscle groups tested on both sides	MMRC 10‐sum score	MMRC new 10‐sum score	MMRC 14‐sum score	Rasch‐built MMRC 10‐sum score
Upper limbs				
Shoulder abductors	+	+	+	+
Elbow flexors	+	+	+	+
Elbow extensors	+	+	+	+
Wrist extensors	+	+	+	+
Wrist flexors	+		+	+
Finger flexors		+	+	
Finger extensors at metacarpophalangeal joints		+	+	
Thumb abductor		+	+	
Index finger abductor		+	+	
Lower limbs				
Hip flexors	+		+	+
Knee flexors	+		+	+
Knee extensors	+		+	+
Foot dorsal flexors	+	+	+	+
Foot plantar flexors	+	+	+	+
Total score^a^	0‐100	0‐100	0‐140	0‐60

Abbreviations: +, muscle groups tested; MMRC, Modified Medical Research Council.

a MMRC 10‐sum score and MMRC new 10‐sum score, MMRC 14‐sum score: each muscle group is scored from 0 (paralysis) to 5 (normal strength), Rasch‐built MMRC 10‐sum score: each muscle group is scored from 0 (paralysis) to 3 (normal strength)—a higher value indicates better muscle strength.

Secondary efficacy outcomes included the MMRC new 10‐sum score developed specifically for this study, the Rasch‐built MMRC 10‐sum score,^7^ the MMRC 14‐sum score (Table 1), grip strength measured with a dynamometer for the most affected hand and disability in daily activities, as assessed with the inflammatory neuropathy course and treatment (INCAT) disability score^8^ as used in MMN by Stangel et al.^9^ Total score on this scale ranges from 0 (no signs of disability) to 10 (most severe disability score) and the upper‐limb subscore ranges from 0 to 5.

Throughout the study, safety was evaluated by assessing the occurrence of adverse events (AEs) and their relationship to the product. A physical examination was performed and vital signs, biochemical, and haematological parameters were also monitored.

### 2.4 Statistics

Sample size for the non‐inferiority test comparing IqYmune® and Kiovig® was calculated based on the primary criterion of MMRC 10‐sum score, assuming no difference between treatments. A difference ≤2 points in MMRC 10‐sum score between the two products was considered not to be clinically meaningful. With a non‐inferiority margin of 2, a within‐participant error variance of 2.5, a one‐tailed α risk of 2.5% and 90% power, 16 evaluable participants were required to achieve a lower confidence interval (CI) boundary ≥−2 for the IqYmune®‐Kiovig® difference, assuming a true difference of 0.

The analysis of efficacy endpoints was based on a linear mixed model, to obtain a 95% CI for the difference between IqYmune® and Kiovig®. The model included subject as a random effect, and product (IqYmune®/Kiovig®), period (1/2) and sequence (A/B) as fixed effects, with baseline value (ie, before the start of the first period) as a continuous fixed‐effect covariate.

The non‐inferiority of IqYmune® relative to Kiovig® was assessed in one‐tailed tests with a nominal α risk of 2.5%. Non‐inferiority was considered to be demonstrated if the lower limit of the 95% CI of the difference (IqYmune® − Kiovig®) was greater than −2.

The total treated set (TTS) was defined as all subjects receiving at least one dose of product. The modified intent‐to‐treat (mITT) population was defined as all randomised subjects receiving at least one administration of product, for whom a baseline level and at least one post‐treatment MMRC efficacy assessment were available. The *per protocol* set (PPS) was defined as all subjects from the mITT population completing the protocol without deviation (as assessed during the protocol deviation review meeting before unblinding) likely to affect the statistical analysis.

For efficacy evaluations, the mITT population was used for the primary analysis and the *per‐protocol* set was used for the secondary analysis. Sensitivity analyses were also performed on these populations. The TTS was defined as all subjects who received at least one dose of investigated medicinal product. The TTS was used for all safety analyses.

## 3 RESULTS

### 3.1 Participant characteristics and treatment

**Figure 1:**
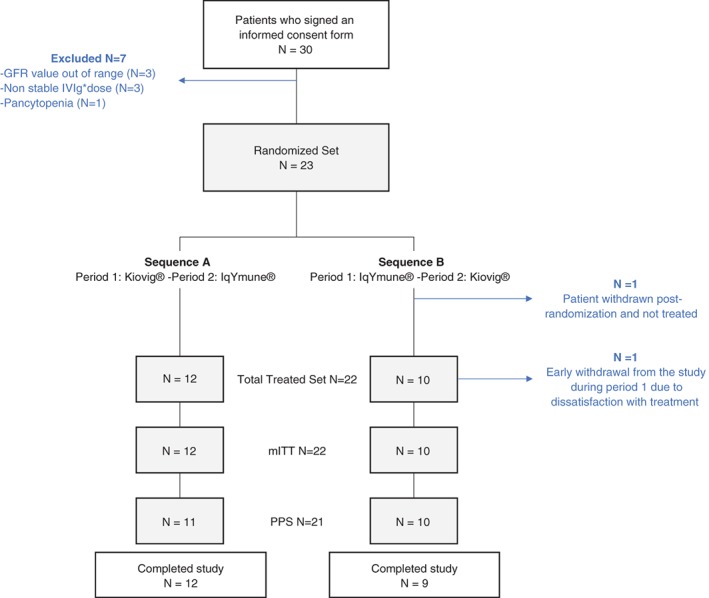
Participants disposition. GFR, glomerular filtration rate; IVIg, intravenous immunoglobulin; mITT, modified intent‐to‐treat; PPS, per protocol set

Between October 2013 and July 2015, 30 participants were screened, and 23 participants were randomised to sequence A (Kiovig® then IqYmune®; N = 12) or B (IqYmune® then Kiovig®; N = 11). One participant randomised to sequence B was excluded before the first dose administration because the previous IVIg dose was not stable. Twenty‐two participants received at least one course of product. One participant in sequence B withdrew his consent 4 months after treatment initiation, due to dissatisfaction with study treatment. This participant was not excluded from any of the populations for analysis. A flow chart summarising the distribution of the participants is provided in Figure 1.

The baseline characteristics of the patients were similar in the two groups (Table 2). Most of the participants were men. Median age was 48.0 years, and there was one patient over the age of 75 years. Sixteen of the 22 participants (72.7%) had at least one relevant concomitant disease in their medical or surgical history. Vascular disorders were the most frequent and were found in seven (31.8%) participants. All participants had already been on a stable dose of IVIg therapy for MMN for at least 3 months before inclusion in the study. The median time from initial diagnosis to entry into this study was 4.3 years.

**Table 2:** Baseline demographic and clinical characteristics of the participants with MMN

	Sequence A^d^ N = 12	Sequence B^d^ N = 10
Male: female ratio, n (%)	11 (91.7):1 (8.3)	8 (80.0):2 (20.0)
Age (y)		
Median (min, max)	47.0 (32.0, 78.0)	49.0 (31.0, 64.0)
BMI (kg/m^2^)^a^		
Median (min, max)	26.3 (18.1, 32.8)	24.7 (22.2, 35.1)
European Federation of Neurological Societies/Peripheral Nerve Society diagnostic criteria, n (%)		
Definite	11 (91.7)	8 (80.0)
Probable	1 (8.3)	2 (20.0)
Time since first symptoms (y)^b^		
Median (min, max)	5.4 (1.0, 18.0)	8.3 (1.8, 20.5)
Time since diagnosis (y)^b^		
Median (min, max)	3.4 (0.4, 9.8)	4.7 (1.2, 20.5)
Prior treatment of MMN other than IVIg since diagnosis, n (%)		
Immunosuppressive	1 (8.3%)	3 (30.0%)
Other^c^	1 (8.3%)	0
MMRC 10‐sum score		
Median (min, max)	97.5 (84.0, 100.0)	96.0 (60.0, 100.0)
MMRC new 10‐sum score		
Median (min, max)	93.0 (63.0, 97.0)	95.0 (45.0, 99.0)
Rasch‐built MMRC 10‐sum score		
Median (min, max)	58.0 (47.0, 60.0)	56.5 (34.0, 60.0)
MMRC 14‐sum score		
Median (min, max)	133.0 (98.0, 137.0)	135.0 (74.0, 139.0)
Total INCAT disability score		
Median (min, max)	2.0 (1.0, 6.0)	3.5 (0.0, 5.0)
Normalised grip strength (%)		
Median (min, max)	60.0 (0.0, 130.0)	72.0 (6.0, 115.0)

Abbreviations: BMI, body mass index; IVIg, intravenous immunoglobulin, MMN, multifocal motor neuropathy.

Modified Medical Research Council (MMRC) 10‐sum score and MMRC new 10‐sum score (range 0‐100), MMRC 14‐sum score (range 0‐140), Rasch‐built MMRC 10‐sum score (range 0‐60), a higher value indicates better muscle strength; Total INCAT disability score (range 0‐10), a higher value indicates maximal disability.

a BMI = weight (kg)/height (m)^2^.

b Time derived as: (screening date − event date)/365.25.

c Participant was treated with gabapentin, chloraminophene, cetirizine, and prednisolone.

d In sequence A, participants were treated first with Kiovig® for 21 to 25 weeks (period 1) then with IqYmune® for 21 to 25 weeks (period 2). In sequence B, participants were treated first with IqYmune® and then with Kiovig®.

### 3.2 Efficacy

Sensitivity analysis and analysis of efficacy endpoints were performed on the mITT and PPS populations (Figure 1).

#### 3.2.1 Primary efficacy criterion: Mean MMRC sum score during the evaluation period

The comparison test was based on a linear‐mixed model estimating the effect of product, period, and sequence. The estimates of the effects of each of these three factors were adjusted for the other two factors and for baseline MMRC 10‐sum score.

**Figure 2:**
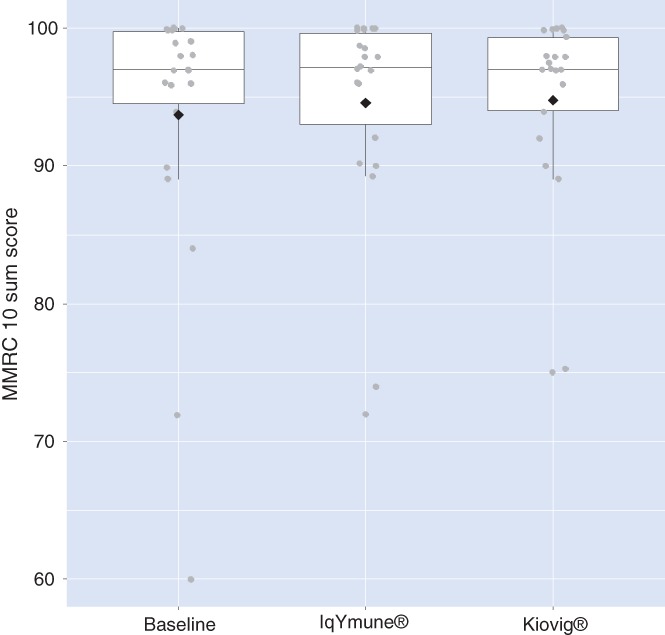
Geometric boxplots of modified Medical Research Council (MMRC) 10‐sum scores at baseline and 6 months after each treatment sequence. Modified intent‐to‐treat, mITT population. The lower limit of a box represents the first quartile, that is, 25% of data lie below this value; the upper limit of the box represents the third quartile, that is, 25% of the data lie above this value; the horizontal line within the box indicates the median, that is, 50% of data lie above this value. The diamond represents the mean value of the distribution, and the dots outside the box represent outliers. This figure shows that the medians after IqYmune® treatment and Kiovig® treatment are similar to the baseline value and that the distributions are not significantly different

No sequence or period effect was detected. The non‐inferiority of IqYmune® relative to Kiovig® was demonstrated: the estimated difference between IqYmune® and Kiovig® was −0.01, with a 95% CI of [−0.51, 0.48] for the mITT population (Figure 2) and −0.14 [−0.60, 0.31] for the PPS (Table 3). The associated *P*‐values for non‐inferiority were <0.0001.

**Table 3:** Primary efficacy outcome‐estimated means of MMRC 10‐sum score 13 to 26 weeks after the start of administration for each product, linear mixed model—mITT and PPS

Population	Covariate	Least square means: estimate [95% CI]	Differences: estimate [95% CI]	*P* value
mITT N = 22	Product			
	IqYmune®	94.5 [93.5, 95.6]	−0.01 [−0.51, 0.48]	0.96
	Kiovig®	94.5 [93.6, 95.5]		
	Period^a^			
	1	94.4 [93.4, 95.4]	−0.24 [−0.73, 0.25]	0.32
	2	94.7 [93.7, 95.6]		
	Sequence^a^			
	A	95.0 [93.9, 96.0]	0.90 [−0.85, 2.65]	0.30
	B	94.1 [92.58, 95.61]		
Non‐inferiority IqYmune® vs Kiovig®				<0.001
PPS N = 21	Product			
	IqYmune®	94.4 [93.3, 95.6]	−0.14 [−0.60, 0.31]	0.51
	Kiovig®	94.6 [93.6, 95.5]		
	Period^a^			
	1	94.3 [93.2, 95.5]	−0.37 [−0.83, 0.08]	0.10
	2	94.7 [93.7, 95.6]		
	Sequence^a^			
	A	94.9 [93.8, 96.0]	0.80 [−1.06, 2.67]	0.38
	B	94.1 [92.5, 95.7]		
Non‐inferiority IqYmune® versus Kiovig®				<0.001

Abbreviations: CI, confidence interval; MMRC, Modified Medical Research Council.

Range of mean MMRC 10‐sum score result: 0 (complete paralysis) to 100 (full strength). Modified‐intent‐to‐treat (mITT), and per protocol set (PPS) populations.

a In sequence A, participants were first treated with Kiovig® for 21‐25 weeks (period 1) then with IqYmune® for 21‐25 weeks (period 2). In sequence B, participants were first treated with IqYmune® then with Kiovig®.

#### 3.2.2 Secondary efficacy criteria

Using the same linear‐mixed model as for the primary endpoint, no statistically significant difference between IqYmune® and Kiovig® was detected for the MMRC new 10‐sum score, the Rasch‐built MMRC 10‐sum score, the MMRC 14‐sum score, total INCAT disability score or normalised grip strength measured during the evaluation period (Table 4). The improvement in clinical global impression (CGI) measured at the end of each period was also similar for the two products, with no specific change observed in more than half of the participants (Table 5).

**Table 4:** Secondary efficacy outcome assessments during the 13 weeks after the initiation of treatment with each product, linear mixed model—mITT population

Covariate: product	Least square means: estimate [95% CI]	Differences: estimate [95% CI]	*P* value
MMRC new 10‐sum score			
IqYmune®	88.8 [87.2, 90.4]	0.15 [−0.56, 0.85]	0.67
Kiovig®	88.7 [87.3, 90.1]		
Rasch‐built MMRC 10‐sum score			
IqYmune®	55.9 [55.3, 56.4]	0.12 [−0.22, 0.46]	0.46
Kiovig®	55.7 [55.2, 56.3]		
MMRC 14‐sum score			
IqYmune®	127.6 [125.6, 129.7]	0.10 [−0.65, 0.85]	0.79
Kiovig®	127.5 [125.8, 129.3]		
Total INCAT disability score			
IqYmune®	2.5 [2.21, 2.74]	−0.03 [−0.29, 0.23]	0.7974
Kiovig®	2.5 [2.32, 2.71]		
Normalised grip strength (%)			
IqYmune®	52.3 [47.0, 57.7]	−1.53 [−5.80, 2.73]	0.46
Kiovig®	53.9 [49.4, 58.3]		

Abbreviations: CI, confidence interval

Modified Medical Research Council (MMRC) new 10‐sum score (range 0‐100), Rasch‐built MMRC 10‐sum score (range 0‐60), MMRC 14‐sum score (range 0‐140), a higher value indicates better muscle strength; Total INCAT disability score (range 0‐10), a higher value indicates maximal disability. Modified‐intent‐to‐treat population (mITT, N = 22).

**Table 5:** Clinical global impression, by product, measured at the end of each evaluation period

	IqYmune® N = 22	Kiovig® N = 21
Rate of global improvement		
1 = Very much improved	0	0
2 = Much improved	3 (13.6)	2 (9.5)
3 = Minimally improved	5 (22.7)	5 (23.8)
4 = No change	12 (54.5)	12 (57.1)
5 = Minimally worse	2 (9.1)	2 (9.5)
6 = Much worse	0	0
7 = Very much worse	0	0

Data are numbers (%) of participants.

### 3.3 Safety

The population used for the evaluation of safety consisted of the 22 participants who received at least one infusion of product. Participant exposure levels were similar for IqYmune® and Kiovig® (Table 6).

**Table 6:** Participant exposure to the medicinal product

	IqYmune® N = 22	Kiovig® N = 21
Duration of IMP exposure (d)	169.0 (64.0, 176.0)	169.0 (164.0, 180.0)
Course frequency (wk)	4.0 (3.9, 8.3)	4.0 (4.0, 8.6)
Total number of courses	6.0 (2.0, 6.0)	6.0 (3.0, 6.0)
Total number of infusions	12.0 (6.0, 20.0)	12.0 (6.0, 20.0)
IMP dose per course (g/kg)	1.5 (1.0, 2.0)	1.4 (1.0, 2.0)
Cumulative IMP dose (g/kg)	6.3 (3.7, 11.6)	6.5 (3.7, 11.7)
Maximal flow rate over all infusions (mL/kg/h)	2.9 (1.0, 6.0)	3.1 (1.3, 6.0)

Abbreviations: IMP, investigated medicinal product.

The data shown are medians (min, max).

Both products were well‐tolerated. There were no significant differences between IqYmune® and Kiovig® in the number of commonly reported AEs. None of the observed AEs was serious, and all resolved without sequelae. Of the 161 AEs reported in 17 (77.3%) participants, 71 were considered related to the investigated medicinal product, 32 of these events occurring in 11 (52.4%) participants receiving Kiovig® and 39 in 10 (45.5%) participants receiving IqYmune® (Table 7). The most common AEs were headache and fatigue, for both products (Table 7). One participant had a single episode of severe headache attributed to IqYmune®; this episode lasted 2 days and resolved with medication without sequelae.

**Table 7:** Overview of the adverse event (AE) profile in the population used for safety assessments

	IqYmune® N = 22	Kiovig® N = 21
Any AE	83 (16; 72.7%)	78 (15; 71.4%)
Serious AEs	0	0
All drug‐related AEs	39 (10; 45.5%)	32 (11; 52.4%)
Drug‐related AEs occurring in at least two participants with either product		
Headache	14 (6; 27.3%)	14 (9; 42.9%)
Neutropenia	3 (1; 4.5%)	5 (1; 4.8%)
Pruritus	6 (1; 4.5%)	0
Fatigue	3 (2; 9.1%)	1 (1; 4.8%)
Leukopenia	1 (1; 4.5%)	3 (1; 4.8%)
Nausea	2 (2; 9.1%)	1 (1; 4.8%)
Vomiting	2 (1; 4.5%)	1 (1; 4.8%)

The data shown are the numbers of AEs occurring (number and percentage of participants).

Haematological and biochemical parameters before and after each course revealed no signs of haemolysis or renal impairment with either Kiovig® or IqYmune (data not shown). No thromboembolic events occurred in any of the participants.

## 4 DISCUSSION

This phase III randomised, comparative, active‐control, double‐blind study with a crossover design demonstrated the non‐inferiority both in efficacy and safety of IqYmune® compared with Kiovig® for the maintenance treatment of MMN.

The non‐inferiority margin of two points for MMRC 10‐sum score mean between the two products was considered adequate. MMN is a rare, slowly progressing disease. A crossover design was also considered appropriate for this study. One weakness of crossover trials is the carryover effect across periods. The 12 weeks during which no assessments were made at the start of each period were considered to constitute an adequate wash‐out phase to prevent carryover effects from previous IVIg treatment.

The non‐inferiority of IqYmune® treatment was established by direct comparison with an active control of proven efficacy. The comparator, Kiovig® has been shown to be more effective than placebo for the maintenance therapy of MMN^7^, ^10^ and has had the European Medicines Agency approval since 2011 and the Food and Drug Administration approval since 2012. In the double‐blind placebo‐controlled study performed by Hahn, the mean maximal grip strength of the most affected hand of adults with MMN increased slightly on Kiovig® treatment (3.75%) but deteriorated by 31.4% on placebo (*P* = 0.005). Hahn et al, also reported that Guy's Neurological Disability Scale^11^ scores worsened in 35.7% of participants during placebo treatment, but not during Kiovig® treatment. Similarly, in the prospective open‐label, non‐controlled study performed by Cats et al, muscle strength and disability scores remained stable in MMN patients during Kiovig® treatment.^7^

The non‐inferiority of IqYmune® treatment was demonstrated in both the modified intention‐to‐treat and *per protocol* populations. The *PPS* consisted of all participants completing the full course of assigned treatment with no major protocol violations. The adult participants in this study had all been diagnosed with MMN, on the basis of their signs and symptoms, according to the EFNS/PNS 2010 guidelines.^1^ Muscle/grip strength and weakness were assessed with scales previously used in other trials in participants with immune neuropathies^12^ and MMN.^7^ The use of the MMRC 10‐sum score for assessing muscle strength in MMN was approved by the European Medicines Agency during the scientific advice meeting. The MMRC new 10‐sum score focuses more strongly on the upper limbs than the original MMRC 10‐sum score. Based on the results obtained in Cats cross‐sectional study,^13^ this scale, developed by disease experts, includes clinically relevant distal upper limbs muscle commonly affected in MMN and excludes irrelevant lower limb muscles not usually affected in MMN. The MMRC new 10 sum score yielded lower values by 5 to 6 points than the original MMRC score (difference not tested statistically) so that as anticipated it captured more weakness in a pattern considered by experts to be typical of MMN. Participants had a mean INCAT disability scale score of 2.5 points on therapy, demonstrating persistent disability despite treatment. Mean grip strength 2 weeks after the last course of each product was higher than that just before the course concerned, demonstrating ongoing benefit from IVIg treatment (Supporting Information Table S1).

IVIg has been proven to improve weakness and disability in patients with MMN and is the gold standard treatment of this disabling disease. However, the effect of IVIg on motor symptoms and signs may decline after several years.^4^ Consequently, other therapeutic options are being investigated. Subcutaneous immunoglobulin has been tested in small studies in MMN^14^, ^15^, ^16^, ^17^; although maintenance was not obtained in all subjects, this option could be more convenient for some patients, as for chronic inflammatory demyelinating polyneuropathy.^18^ The addition of eculizumab, which neutralises human complement C5, to IVIg in one small‐controlled trial showed a trend towards improvement^19^ which needs to be confirmed in larger studies.

This study was limited by the lack of high‐quality outcome measures covering all the domains of disability, impairment, and quality of life in MMN.^20^ The outcome measures used in MMN trials still lack standardisation and sensitivity.^21^ At the time the protocol for this trial was designed, there was no validated functional disability scale for MMN. The Rasch‐built overall disability scale for MMN (MMN‐RODS) does overcome the shortcomings of ordinal scales was proposed in 2015^22^ but still needs to be validated in new series.

In summary, IqYmune® was not inferior to Kiovig® in efficacy for the maintenance treatment of MMN. Safety results for both products were consistent with the known safety profile of IVIg. This IqYmune® is a valid option for the maintenance treatment of MMN.

## Supporting information


**Table S1** Changes in mean grip strength (absolute and normalised values) 2 weeks after the last course compared to the score just before this specific course—mITT as treated
