# Supplementary material for: IqYmune® is an effective maintenance treatment for multifocal motor neuropathy: A randomised, double‐blind, multi‐center cross‐over non‐inferiority study vs Kiovig®—The LIME Study
Source: J Peripher Nerv Syst. 2018 Dec 11;24(1):56–63. doi: 10.1111/jns.12291 (PMC6590491; doi:10.1111/jns.12291)
Supplement: Supplementary file 1 — Table S1 Changes in mean grip strength (absolute and normalised values) 2 weeks after the last course compared to the score just before this specific course—mITT as treated [file JNS-24-56-s001.docx]

Table S1: Changes in mean grip strength (absolute and normalized values) 2 weeks after the last course compared to the score just before this specific course - mITT as treated

|  | **Kiovig N=21** | **IqYmune N=22** |
| --- | --- | --- |
| **Changes in absolute values (kPa)** |  |  |
| n | 16 | 17 |
| Median | 0.8 | 2.3 |
| Min , Max | -12.0 , 38.7 | -8.7 , 22.0 |
| **Changes in normalized values (%)** |  |  |
| n | 16 | 17 |
| Median | 0.5 | 2.0 |
| Min , Max | -9.0 , 27.4 | -6.9 , 20.8 |
